# *Death by Strychnine*—Report on the Case of the Late Dr. W. C. Warner

**Published:** 1847-05

**Authors:** 


*Death by Strychnine*—Report on the case of the late Dr. W. C. Warner.—At a late meeting of the Addison County Medical Society of Vermont, the undersigned were appointed a committee to ascertain the facts in the case of one of their members, the unfortunate William Cullen Warner, M. D., of Bristol, who deceased, suddenly, at Montpelier, October 11th, 1846, in the thirty-ninth year of his age, while he was a member of the Legislature.

On account of there having been considerable discrepancy in the published reports in relation to this melancholy event, the committee addressed letters of inquiry to the Hon. Daniel O. Onion, M. D., of the Vermont Senate, and to Charles W. Horton, M. D., Member of the House, each of whom, they had learned, were present during most, if not all, the period of the sudden and tragical event. To the inquiries of the committee, each of these gentlemen has given prompt and satisfactory replies, which in substance are here subjoined.

1. In your opinion how much sulphate of strychnia was taken?

To this Dr. Onion answers, "I think probably from one-fourth to one-half a grain. As he intended to take, and supposed he was taking, morphia, he would be likely to use the same quantity he was in the habit of using of that article, although there was no evidence at the time of the quantity taken." To Dr. Horton, who was called into the room immediately after the accident, Dr. Warner said, "Dr., I have taken by accident an over dose of morphine; help me if you can," at the same time handing him the phial enveloped in paper.

2. How soon after was any effect produced?

Dr. Horton says, "It is my opinion, from facts subsequently obtained from Gen. W. Nash, who occupied the same room with him, that he felt the effects in less than five minutes."

3. What was the first symptom?

Dr. H. replies, "constriction of the throat and tightness of the chest, with rigidity of the muscles in attempting to move." Dr. O. says, "He first complained of a want of air, and requested the window to be raised; whether it was from faintness or a constriction about the respiratory organs, I do not know, although I think the latter."

4. What symptoms ensued from the first till death occurred?

Says Dr. O., "When I first saw him, he was lying upon the bed in a complete *tetanic convulsion;* his head somewhat drawn back; his countenance completely livid, with some frothy matter issuing from his mouth, with frequent moans. The palpebra constantly in motion. This first paroxysm may have lasted some five minutes, which was succeeded by an interval of partial calm." "During this interval," continues Dr. O., "it was somewhat difficult for him to articulate with distinctness. He made several attempts to vomit in this interval, by exciting the fauces with his finger. There seemed to be some constriction about the throat, as it was difficult for him to swallow." "This interval lasted perhaps five minutes, when another paroxysm commenced by a little starting and stiffening of the extremities, and immediately the whole body was thrown into a tetanic paroxysm, in appearance like the first, and lasted two or three minutes, when death ended the struggle."

"In about three minutes from the first paroxysm," says Dr. H., "the tetanus again returned, and in the space of two minutes death closed the scene, with terrible spasms of the entire system. The pulse remained unaffected till the last struggle. It is my opinion that the immediate cause of death was suspension (?) from spasm."

"His appearance," says Dr. O., "led me to believe that death ensued from asphyxia or suffocation. There must have been great congestion of the brain, which of itself might have proved fatal."

5. How soon after taking the article did death occur?

Dr. H. says, "From the best information which I could obtain, I should judge that death ensued in fourteen minutes." "The time from taking the article till death ensued," Dr. O. remarks, "could not have been over twenty minutes."

6. Did his mind remain clear till the last struggle?

"I think," replies Dr. H., "that he was perfectly conscious from the first to the last, except in the paroxysm of tetanus, from the following facts:—1. His appeal which he made to me, as noted in the first article. 2. On loosening his cravat, he requested me to unbutton his vest, at the same time desiring me to take out his gold watch and take care of it. 3. An emetic having been administered, he applied his finger to his throat to provoke a nausea. 4. And, from the last words he uttered, '*I fear, I fear, O God deliver me.*'"

7. What means were used to prevent the fatal result?

Dr. H. says, "On witnessing the first symptoms, I left the room for the purpose of obtaining medicine. I procured an emetic of sulphate of copper and ipecac.; but returning and finding him in a tetanus, I immediate dashed cold water on his head, face and breast, and used the most powerful friction on the extremities. He returned to a state of perfect consciousness. I then proceeded forthwith to administer the emetic, making use of diluents copiously. I sent a messenger for some vinegar and ground mustard, and another for a stomach pump. I used the ground mustard, in warm water freely, to all of which the patient submitted, seeming to be very grateful for the efforts which I was making for his relief. The means were used without any apparent effects." "When death had ensued, a number of the medical fraternity being present, we retired into an adjoining room, when the fatal bottle was produced, with the wrapper still around it. On removing this, it was found labelled 'strychnine.'" Dr. O. states, that "till this time, we were in ignorance of what he had taken." Dr. H. avers, "that here I wish definitely to state, that before the last paroxysm came on, I was fully convinced in my own mind that the fatal drug was not morphia, but strychnia, and I so declared to those present at the time."

From facts before the committee, derived from reliable sources, it appears that on the afternoon of the second day before the fatal accident, Dr. Warner called at an apothecary store in Montpelier, and asked for and purchased what he supposed to have been a bottle of sulphate of morphia. This was handed to him by the apothecary enveloped in a brown paper and twisted at both ends. That on the fatal morning Dr. W. tore off the envelope surrounding the mouth of the bottle, and took a portion of what he supposed to have been morphia. He then proceeded to pour some of the supposed morphia into a small phial in which he had been in the habit of carrying sulphate of morphia, when he was suddenly arrested by the symptoms narrated. It is quite clear that he never entertained any idea of the fatal drug he had taken. "I am certain," says his afflicted brother, "that he never for a moment suspected that he had taken strychnia, and was wholly unconscious of the agency which had produced his awfully unprecedented sufferings."

Dr. W. had never possessed very firm health, and for about two years before his death he had suffered from an inordinate action of the heart, for which he had occasionally taken morphia. This affection of the heart had been the sequence of an inflammatory affection of the chest, which he had early in the year 1844.

The committee have taken considerable pains to ascertain the facts in this melancholy instance of death from a mysterious mistake. The mistake was certainly a singular and mysterious one, both in relation to the apothecary and the unfortunate man. It appears that Dr. W. asked for sulphate of morphia; the apothecary intended and supposed he had sold him morphia till after the fatal event, when he found, through mistake, he had given him, enveloped in a paper, a bottle of sulphate of strychnia in lieu of morphia. This exposition of facts appears to be demanded in justice to the character of the deceased, to the apothecary and to the medical profession.

In a medical point of view, the case is one of much and deep interest, since it so clearly manifests the true and energetic character of this somewhat new medicinal agent. And in a medico-legal consideration, it may prove of immense importance. In the suddenness of the effects, and in the quickness of the fatality, from the use of strychnia, this case is probably without a precedent. Christison, Pereira, and several monographical writers, in the periodicals, have recorded some bad results, and some fatal cases, from over dosing with this agent; but no instance has fallen under our notice in the human subject in which its administration, either accidentally or otherwise, has so speedily and terrifically proved fatal.

"No poison," says Christison, "is endowed with more destructive energy than strychnia." "I have," he adds, "killed a dog in two minutes with the sixth part of a grain, injected in the form of an alcoholic solution into the chest. I have seen a wild boar killed in the same manner with a third of a grain, in ten minutes; and there is little doubt that half a grain thrust into a wound might kill a man in less than a quarter of an hour. It acts in whatever way it is introduced into the system, but most energetically when injected into the veins."

With the exception of prussic and oxalic acids, there is probably no agent possessing an equally destructive power. Strong prussic acid is well known to be sufficiently energetic to destroy cats or dogs, when properly administered, in less than a minute. And Pereira examined the body of a man who had accidently taken oxalic acid in lieu of Epsom salts, and died in twenty minutes.

JONATHAN A. ALLEN, M. D.,
ERASMUS D. WARNER, M. D.,
WM. P. RUSSELL, M. D.
*Boston Med. and Surg. Jour.*

